# Emergence of *BCR*–*ABL1* Fusion in AML Post–FLT3 Inhibitor-Based Therapy: A Potentially Targetable Mechanism of Resistance – A Case Series

**DOI:** 10.3389/fonc.2020.588876

**Published:** 2020-10-20

**Authors:** Ahmad S. Alotaibi, Musa Yilmaz, Sanam Loghavi, Courtney DiNardo, Gautam Borthakur, Tapan M. Kadia, Beenu Thakral, Naveen Pemmaraju, Ghayas C. Issa, Marina Konopleva, Nicholas J. Short, Keyur Patel, Guilin Tang, Farhad Ravandi, Naval Daver

**Affiliations:** ^1^The Department of Leukemia, MD Anderson Cancer Center, Houston, TX, United States; ^2^The Department of Hematopathology, MD Anderson Cancer Center, Houston, TX, United States

**Keywords:** FLT3, BCR-ABL, FLT3 inhibitors, secondary mutations, AML

## Abstract

Despite the promising result with FLT3 inhibitors in AML, the emergence of resistance poses a significant challenge, leading to a shorter response duration and inferior survival. This is frequently driven by on-target or parallel prosurvival mutations. The emergence of *BCR–ABL1* as a mechanism of possible clonal evolution in relapsed AML has rarely been reported. Here we report our experience with three patients who had emergent *BCR–ABL1* fusion at relapse after FLT3 inhibitors–based therapies. The first patient was refractory to multiple lines of therapies, including FLT3 inhibitors–based therapy. Patients 2 and 3 showed some response to combined FLT3-inhibitor and *BCR*–*ABL* targeted therapy (gilteritinib and ponatinib). The availability of effective targeted therapies for *BCR–ABL1* makes this an important aberration to proactively identify and possibly target at relapse post–FLT3-inhibitor therapies.

## Introduction

The development of multiple small-molecule kinase inhibitors targeting *FLT3* has improved the outcome of *FLT3*-mutated acute myeloid leukemia (AML) (1). Despite high response rates with FLT3 inhibitor–based therapies, the emergence of new mutations frequently drives resistance, and resulting in short durations of response and survival (2–5). These emergent mutations may involve the activating loop or gatekeeper residues of the *FLT3* (on target resistance) (2, 3, 5) or genes regulating parallel prosurvival signaling pathways such as PI3K/AKT and RAS/MAPK (off-target resistance) (4, 5). Herein, we report the cases of three patients who relapsed following an FLT3 inhibitor–based therapy, with an emergent *BCR–ABL1* fusion, rendering a potentially targetable mechanism of resistance.

## Clinical Summary

### Patient 1

A 33-year-old woman was diagnosed with AML with a normal karyotype (no molecular testing done locally at baseline). She received 7 + 3 induction without a response and was reinduced with fludarabine and cytarabine (FLAG) with complete remission (CR), followed by four cycles of high-dose cytarabine (HiDAC) consolidation. She relapsed 5 months after the last consolidation and was referred to our institution following an unsuccessful salvage attempt with FLAG.

**FIGURE 1:**
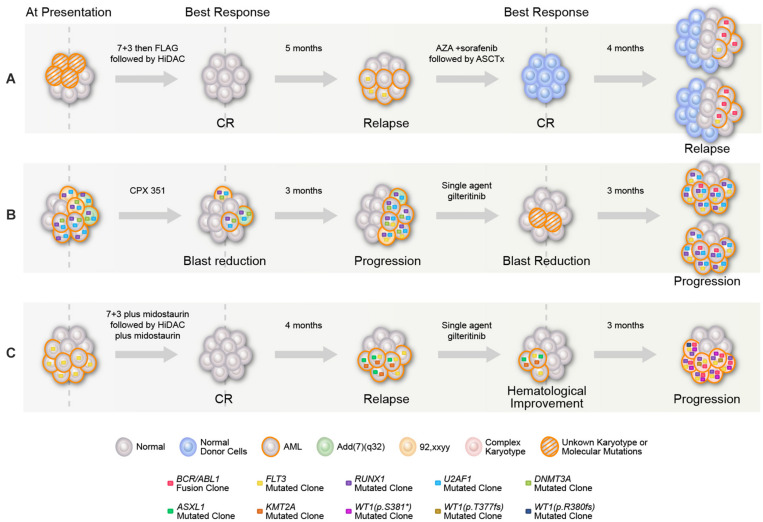
Graphical representation of mutational acquisition/expansion. **(A)** Patient 1, **(B)** patient 2, and **(C)** patient 3. Clonal size was estimated based on variant allelic frequency (VAF) for mutations detected by NGS, allelic ratio for *FLT3*, percentage of *BCR–ABL* to *ABL* transcripts, and number of aberrant metaphases in karyotype. Clones could have coexisted or be mutually exclusive, mutations with high clonal size at the same given point of time were considered to be coexisted, for other mutations, the two possibilities were represented. At last relapse/progression in **(A,B)**, the two branch graphs represent the possibility of coexistence or mutually exclusivity of *FLT3* mutation and *BCR/ABL.*

Our initial bone marrow biopsy revealed 60% blasts, normal karyotype, *FLT3*–ITD (allelic ratio 0.33), with no *BCR–ABL1* fusion or *NPM1*, *RAS*, *IDH1&2*, or *KIT* mutations. The patient received azacitidine with sorafenib with marrow remission after four cycles and underwent allogeneic stem cell transplant (ASCT), but relapsed 60 days post-ASCT with 58% bone marrow blasts, normal karyotype, and *FLT3*–ITD (allelic ratio 0.02). After achieving a short-term remission with idarubicin, cytarabine, and sorafenib combination, subsequent relapse bone marrow demonstrated *t*(9,22) in 7/20 metaphases with a positive *BCR–ABL1* fusion transcript (55%) and *FLT3*–ITD (allelic ratio 0.12) (Figure 1A). The patient died after not responding to phase I agent BP-1001 (L-Grb-2 antisense oligonucleotide NCT01159028) (6).

### Patient 2

A 47-year-old man was diagnosed with AML with [add (7) (q32)], and *DNMT3A*, *RUNX1*, and *U2AF1* mutations on a myeloid NGS panel [no *FLT3* ITD/tyrosine kinase domain (TKD) mutation] at diagnosis at a local institution. He received consecutive therapies with CPX-351, decitabine, and FLAG-IDA, without a response. Repeat bone marrow analysis revealed an emergent *FLT3*–ITD mutation (allelic ratio not known). After receiving a single-agent gilteritinib salvage therapy for 2 months, he was referred to our institution with progressive disease. The initial bone marrow revealed 70% blasts, normal karyotype [no *t*(9,22) detected], positive for *BCR–ABL1* rearrangement (2.8% by FISH) and *BCR–ABL1* fusion (1.92%), *FLT*-3 ITD (allelic ratio 0.49), *RUNX1*, *U2AF1*, and *KRAS* (Figure 1B) (7). The patient did not respond to cladribine, idarubicin, cytarabine, and sorafenib combination, with increased *BCR–ABL1* fusion transcript (7.05%) posttreatment. After failing the next salvage therapy (decitabine, venetoclax, and ponatinib), the patient received CPX-351 plus venetoclax, ponatinib, and gilteritinib combination with a marrow remission (MLFS) (*BCR–ABL1* fusion transcript 0.05%). ASCT was performed with a CR on day 30 post-ASCT (no detectable measurable residual disease by multiparametric flow cytometry, undetectable BCR–ABL1 fusion). The patient died 8 months post-ASCT in CR because of infectious complications.

### Patient 3

A 53-year-old woman with AML, diploid karyotype, and *FLT3*–ITD mutation achieved CR with 7 + 3 plus midostaurin. She relapsed 2 months later with *t*(6,11) (p24,q14), *FLT3*–ITD, *ASXL1*, and *KMT2A (ASXL1, KMT2A* were not detectable at baseline). After failing mitoxantrone, etoposide, and cytarabine combination and then salvage with single-agent gilteritinib, the bone marrow revealed 50% blasts with BCR–ABL1 rearrangement (91% by FISH). Upon referral to our institution, repeat bone marrow biopsy showed 38% blasts, complex cytogenetics with *t*(9,22), *BCR–ABL1* fusion transcript (63.9%), *FLT3*–ITD (allelic ratio 0.48), *RUNX1*, *WT1* (three distinct mutations) (Figure 1C). On day 21 of the salvage therapy (decitabine, gilteritinib, and ponatinib), the patient had MLFS (persistent *BCR–ABL1* at a level of 76.19%) and later died because of infectious complications.

## Discussion

Targeting *FLT3* mutations has improved outcomes in AML. Acquisition/expansion of mutations drives secondary resistance to FLT3-inhibitor therapy. Activation of parallel signaling pathways, such as PI3K/AKT and RAS/MAPK, is an increasingly recognized mechanism of FLT3-inhibitor resistance (2, 4). Targeted NGS at baseline and relapse in 41 relapsed FLT3-mutated patients before and after single-agent gilteritinib demonstrated emergent RAS/MAPK pathway mutations (*NRAS*, *KRAS*, *PTPN11*, and *NF1*) in 15 (36.6%) and newly detectable *BCR/ABL1* fusions in 2 (5%) patients (5).

The Philadelphia chromosome, *t*(9,22) (q34,q11.2), results in a *BCR–ABL1* fusion gene, encoding a constitutively active oncogenic tyrosine kinase. The incidence of the Ph chromosome in *de novo* AML ranges from 0.5 to 3% (8). Acquisition of *BCR–ABL1* as a secondary abnormality and a mechanism of possible clonal evolution in relapsed AML has rarely been reported postchemotherapy treatment (9) and now post–FLT3 inhibitor–based therapy (5, 10).

Although rare (3–5%), identification of *BCR–ABL1* fusion at relapse has clinical significance as it is a targetable mutation. In this report, patients 2 and 3 were refractory to FLT3 inhibitor–based therapies, but eventually responded to combined FLT3-inhibitor and *BCR*–*ABL* targeted therapy (gilteritinib and ponatinib). Patient 1 remained refractory to multiple conventional salvage chemotherapy plus FLT3-inhibitor regimens, possibly in some part due to *BCR*–*ABL*–mediated resistance to FLT3 inhibitor–based therapies. Ponatinib is a potent kinase inhibitor with pan-BCR–ABL1 inhibitor activity and strong FLT3-inhibitor activity. Smith et al. (11) demonstrated *in vitro* activity of ponatinib against FLT3–ITD and F691 gatekeeper mutation. Gilteritinib is a selective FLT3 inhibitor with potent activity against *FLT3–ITD*, as well as *TKD* mutations, although 5 (12.2%) of 42 patients acquired *F691* gatekeeper mutations at relapse post–gilteritinib therapy (5). Combinatorial or sequential use of gilteritinib and ponatinib to overcome each individual drug resistance could be of interest for prospective evaluation, especially in FLT3-mutated patients with detectable subclonal acquisition of *BCR–ABL1* fusion. Combining these two potent FLT 3 inhibitors should ideally be performed under clinical investigation/trial setting, with close monitoring and caution for myelosuppression, ideally in large leukemia centers with significant expertise. An increased understanding of the mechanisms of FLT3-inhibitor resistance may help identify and target known druggable pathways of resistance to overcome primary and secondary resistance in clinical practice.

## Data Availability Statement

All datasets generated in this study are included in the article/supplementary material.

## Ethics Statement

The studies involving human participants were reviewed and approved by the Institutional Review Board (IRB), MD Anderson Cancer Center (MDACC) IRB protocol DR09-0223 and PA12-0395. The patients/participants provided their written informed consent to participate in this study.

## Author Contributions

ND, AA, and MY collected the data, conceived, designed, and wrote the manuscript. SL, KP, BT, and GT analyzed and reported the molecular and cytogenetics data. CD, GB, TK, NP, GI, MK, NS, and FR treated the patients, read, revised, and approved the final manuscript. All authors contributed to the article and approved the submitted version.

## Conflict of Interest

The authors declare that the research was conducted in the absence of any commercial or financial relationships that could be construed as a potential conflict of interest.

## References

[1] Daver N Schlenk RF Russell NH Levis MJ. Targeting FLT3 mutations in AML: review of current knowledge and evidence. *Leukemia.* (2019) 33:299–312. 10.1038/s41375-018-0357-9 30651634 PMC6365380

[2] Daver N Cortes J Ravandi F Patel KP Burger JA Konopleva M Secondary mutations as mediators of resistance to targeted therapy in leukemia. *Blood.* (2015) 125:3236–45. 10.1182/blood-2014-10-605808 25795921 PMC4440880

[3] Cools J Mentens N Furet P Fabbro D Clark JJ Griffin JD Prediction of resistance to small molecule FLT3 inhibitors: implications for molecularly targeted therapy of acute leukemia. *Cancer Res.* (2004) 64:6385–9. 10.1158/0008-5472.CAN-04-2148 15374944

[4] Piloto O Wright M Brown P Kim KT Levis M Small D. Prolonged exposure to FLT3 inhibitors leads to resistance via activation of parallel signaling pathways. *Blood.* (2007) 109:1643–52. 10.1182/blood-2006-05-023804 17047150 PMC1794049

[5] McMahon CM Ferng T Canaani J Wang ES Morrissette JJD Eastburn DJ Clonal selection with RAS pathway activation mediates secondary clinical resistance to selective FLT3 inhibition in acute myeloid leukemia. *Cancer Discov.* (2019) 9:1050–63. 10.1158/2159-8290.CD-18-1453 31088841 PMC11994087

[6] Ohanian M Kantarjian HM Ravandi F Borthakur G Garcia-Manero G Andreeff M Safety, pharmacokinetics, and efficacy of BP-100-1.01 (Liposomal Grb-2 antisense oligonucleotide) in patients with refractory or relapsed acute myeloid leukemia (AML), philadelphia chromosome positive chronic myelogenous leukemia (CML), acute lymphoblasti. *Blood.* (2015) 126:3801 10.1182/blood.v126.23.3801.3801

[7] Luthra R Patel KP Reddy NG Haghshenas V Routbort MJ Harmon MA Next-generation sequencing-based multigene mutational screening for acute myeloid leukemia using MiSeq: applicability for diagnostics and disease monitoring. *Haematologica.* (2014) 99:465–73. 10.3324/haematol.2013.093765 24142997 PMC3943309

[8] Konoplev S Yin CC Kornblau SM Kantarjian HM Konopleva M Andreeff M Molecular characterization of de novo Philadelphia chromosome-positive acute myeloid leukemia. *Leuk Lymphoma.* (2013) 54:138–44. 10.3109/10428194.2012.701739 22691121 PMC3925981

[9] Kurt H Zheng L Kantarjian HM Tang G Ravandi F Garcia-Manero G Secondary philadelphia chromosome acquired during therapy of acute leukemia and myelodysplastic syndrome. *Mod Pathol.* (2018) 31:1141–54. 10.1038/s41379-018-0014-x 29449681

[10] Kasi PM Litzow MR Patnaik MM Hashmi SK Gangat N. Clonal evolution of AML on novel FMS-like tyrosine kinase-3 (FLT3) inhibitor therapy with evolving actionable targets. *Leuk Res Rep.* (2016) 5:7–10. 10.1016/j.lrr.2016.01.002 26904404 PMC4726703

[11] Smith CC Lasater EA Zhu X Lin KC Stewart WK Damon LE Activity of ponatinib against clinically-relevant AC220-resistant kinase domain mutants of FLT3-ITD. *Blood.* (2013) 121:3165–71. 10.1182/blood-2012-07-442871 23430109 PMC3630831

